# Binding and Dynamics Demonstrate the Destabilization of Ligand Binding for the S688Y Mutation in the NMDA Receptor GluN1 Subunit

**DOI:** 10.3390/molecules28104108

**Published:** 2023-05-15

**Authors:** Jake Zheng Chen, William Bret Church, Karine Bastard, Anthony P. Duff, Thomas Balle

**Affiliations:** 1 Sydney Pharmacy School, Faculty of Medicine and Health, The University of Sydney, Camperdown, NSW 2006, Australiabret.church@sydney.edu.au (W.B.C.);; 2 Brain and Mind Centre, The University of Sydney, Camperdown, NSW 2050, Australia; 3 National Deuteration Facility, Australian Nuclear Science and Technology Organization, New Illawarra Road, Lucas Heights, NSW 2234, Australia

**Keywords:** molecular dynamics, NMDA receptor, membrane protein, ligand binding, encephalopathies

## Abstract

Encephalopathies are brain dysfunctions that lead to cognitive, sensory, and motor development impairments. Recently, the identification of several mutations within the *N*-methyl-D-aspartate receptor (NMDAR) have been identified as significant in the etiology of this group of conditions. However, a complete understanding of the underlying molecular mechanism and changes to the receptor due to these mutations has been elusive. We studied the molecular mechanisms by which one of the first mutations within the NMDAR GluN1 ligand binding domain, Ser688Tyr, causes encephalopathies. We performed molecular docking, randomly seeded molecular dynamics simulations, and binding free energy calculations to determine the behavior of the two major co-agonists: glycine and D-serine, in both the wild-type and S688Y receptors. We observed that the Ser688Tyr mutation leads to the instability of both ligands within the ligand binding site due to structural changes associated with the mutation. The binding free energy for both ligands was significantly more unfavorable in the mutated receptor. These results explain previously observed in vitro electrophysiological data and provide detailed aspects of ligand association and its effects on receptor activity. Our study provides valuable insight into the consequences of mutations within the NMDAR GluN1 ligand binding domain.

## 1. Introduction

Early infantile epileptic encephalopathies are some of the most devastating early-onset epilepsies and can lead to cognitive, sensory, and motor development impairments. The etiology of these syndromes is wide-ranging and can be structural, metabolic, neurological, or genetic in nature [1]. Recently, due to advances in high throughput sequencing technologies, there has been an increase in the identification of genetic mutations associated with early infantile epileptic encephalopathies, including mutations in the *N*-methyl-D-aspartate receptor (NMDAR) [2].

**Figure 1:**
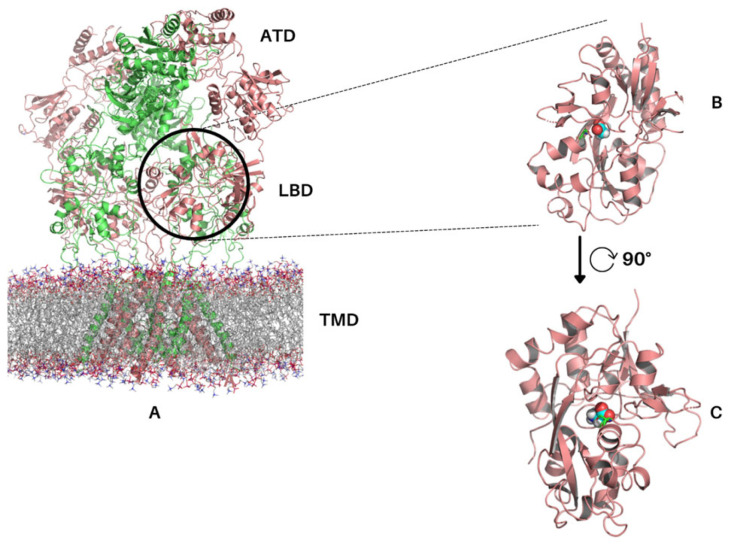
**GluN1/GluN2B receptor (PDB: 6CNA) and GluN1 LBD (PDB: 4NF8).** (**A**) Full NMDA receptor GluN1/GluN2B assembly in a phospholipid membrane. GluN1 is shown in salmon, and GluN2B is shown in green. (**B**,**C**) LBD of GluN1 shown in cartoon representation. Glycine is shown in space fill representation in cyan, and Ser688 is shown in green. Note that the CTD is not visible in the electron microscopy structure shown. Figure generated using PyMOL [44].

NMDARs are ionotropic glutamate receptors (iGluR) and consist of four subunits, which are typically two GluN1 subunits and two GluN2 subunits (Figure 1A); however, the existence of other triheterotetrameric arrangements has been reported [3,4,5]. They are unique in their mode of activation, requiring simultaneous binding of glutamate at the GluN2 subunit and a co-agonist such as a glycine [6], D-serine [7], D-alanine [6,8], or D-cycloserine [7] at the GluN1 subunits [9,10,11], along with membrane depolarisation to displace a Mg^2+^ ion block present in the channel [12,13]. Together, these events activate the receptor and enable Ca^2+^ influx and further downstream effects within the cell [14]. 

Structurally, all GluN subunits consist of an amino terminal domain (ATD), a ligand binding domain (LBD), a transmembrane domain (TMD), and an intracellular carboxyl-terminal domain (CTD) (Figure 1A). X-ray structures of the NMDAR GluN1 LBD were originally reported in 2003 [7], and advances in cryogenic electron microscopy have since led to the membrane and extracellular receptor structures being solved [15,16,17,18,19]. Significantly aided by crystallographic work [20], the individual domains appear largely physically discreet. The NMDAR is normally thought to be involved in learning and memory formation [21,22]. However, dysfunction of the NMDAR has been associated with complex, multifactorial conditions such as schizophrenia [23,24,25,26] and Alzheimer’s disease [27,28]. There have been several studies conducted which utilize molecular dynamics simulations to study the function and effects of the NMDAR [29,30,31,32,33].

Generally, mutations within the GluN1 subunit are associated with conditions such as hypotonia [34], hyperkinetic and stereotyped movements, as well as other conditions [35,36]. Predominantly, mutations in the GluN1 subunit have been identified within the TMD [33,34,35,36,37,38,39,40,41,42,43]; however, mutations within the ATD have also been reported [36]. The first mutation within the LBD linked to infantile epileptic encephalopathies, Ser688Tyr (S688Y), was described by Zehavi et al., in 2017 [34]. Skrenkova et al. [33] established the effects of the S688Y mutation in terms of a significant decrease in functional potency for both glycine and D-serine along with a differential regulation in surface delivery of the mutant NMDAR in HEK293 cells.

However, the molecular mechanisms by which the S688Y mutation leads to decreased potency are yet to be established. We utilize computational methods, including a combination of docking and molecular dynamics simulations, to assist in understanding the ligand dynamics within the binding site. The binding thermodynamics relevant to protein–ligand binding was also estimated in the study. Computer-aided drug design (CADD) utilizes these techniques to determine the function and structure of the protein as a part of structure-based drug design (SBDD) and alchemical methods to determine ligand binding affinity as a part of ligand-based drug design (LBDD).

Our results reveal how the receptor functions and, specifically, how the S688Y mutation leads to impaired receptor function. These calculations demonstrate changes in the receptor–ligand complex structure with important concomitant alterations of the electrostatic and water-bridge network present within the binding site. This gives a deeper insight into the effects of mutations within the NMDAR, specifically those crucial within the LBD and for ligand binding.

## 2. Results

### 2.1. Ligand Docking to GluN1 LBD

**Figure 2:**
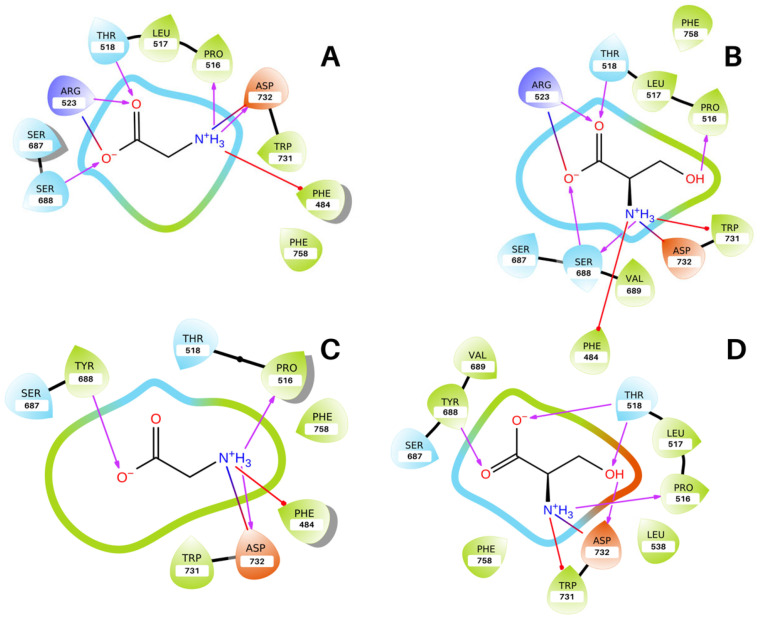
**2D interactions diagram of ligands bound to GluN1 LBD.** Hydrophobic residues are shown in green, polar residues are shown in cyan, positively charged residues are shown in purple, and negatively charged residues are shown in orange. Hydrogen bonds are shown as purple arrows, π-cation interactions are shown as red lines, and salt bridges are shown as red-blue lines. The tip of the tear-drop shape points towards the side chain of the residue; dots on a connection indicate a residue not making contact with the ligand. (**A**) Glycine bound to the wild-type GluN1 LBD (**B**) D-serine bound to the wild-type GluN1 LBD. (**C**) Glycine bound to the S688Y GluN1 LBD (**D**) D-serine bound to the S688Y GluN1 LBD. Figure generated using the ligand interaction visualization module in Maestro 2021.4 [44,45].

**Figure 3:**
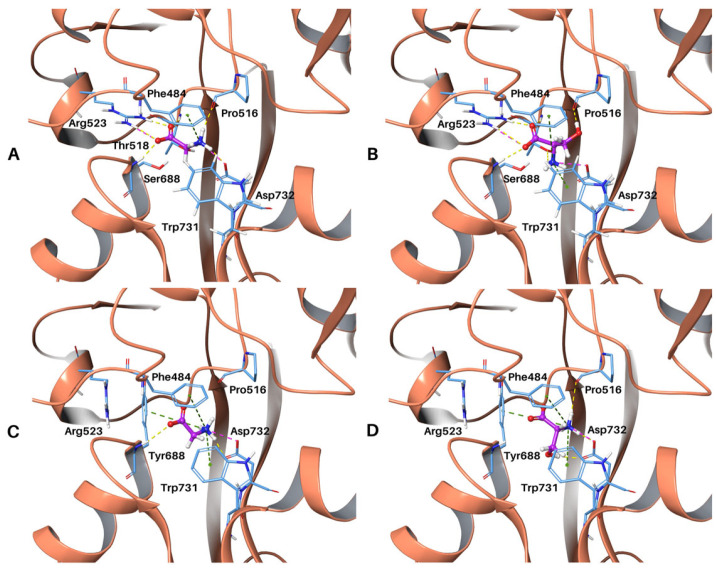
**Top binding poses of glycine and D-serine at the GluN1 LBD (PDB: 4NF8).** Carbon atoms of ligands are shown in magenta, with interacting residues shown in light blue and the ribbon of the receptor shown in salmon. Hydrogen bonds are shown as yellow dashed lines; ionic interactions are shown as purple dashed lines; π-cation interactions are shown as green dashed lines and aromatic hydrogen bonds are shown as cyan dashed lines. (**A**) Glycine bound to wild-type GluN1. (**B**) D-serine bound to wild-type GluN1. (**C**) Glycine bound to S688Y GluN1. (**D**) D-serine bound to S688Y GluN1. Figure generated using PyMOL [44].

Docking of both glycine and D-serine in the wild-type GluN1 LBD (Figure 2A,B and Figure 3A,B) utilizing a flexible receptor-docking algorithm revealed key salt bridge interactions between the carboxylate group of both ligands and the guanidium group of Arg523 of the protein as well as between the ammonium groups and the side chain carboxylate group of Asp732. Further interactions involved π-cation interactions with the phenyl group of Phe484, and D-serine displayed π-cation interactions with Trp731. Hydrogen bonding was also present in the docked conformations with the backbone of residues Pro516, Thr518, Ser688 and side chains of residues Arg523 and Trp732. These interactions are comparable with those observed in the X-ray structures of the NMDAR GluN1 LBD co-crystallized with glycine (PDB ID: 1PB7) and D-serine (PDB ID: 1PB8) by Furukawa and Gouaux [7]. This demonstrates that our docking protocol is valid and appropriate for studying the S688Y mutant. The root mean square deviation (RMSD) of all ligand-heavy atoms for the docked pose when compared to the X-ray structure was 0.8 Å (PDB: 1PB7) for glycine and 1.7 Å for D-serine when superimposed on protein heavy atoms.

In the docked poses, the carboxylate and ammonium groups of glycine and D-serine were present in a polar pocket of the binding site, with the hydroxymethyl group of D-serine present in a hydrophobic sub-pocket. However, in the S688Y mutant receptor, the environment is changed with fewer polar residues present in the environment of both glycine and D-serine due to the alterations of the binding pocket induced because of the change to tyrosine. Furthermore, the key Arg523 residue is not present in the binding pocket due to steric hindrance from the bulky Tyr688 hydroxyphenyl side chain (Figure 3C,D), which upon docking leads to an associated loss of a salt bridge and hydrogen bond in comparison with the wild-type configuration with serine (Figure 2C,D). Other interactions, however, remained intact. Of interest is the main chain nitrogen atom of Tyr688, which continues to facilitate hydrogen bonding with the carboxylate group of glycine and D-serine (Figure 3C,D) with the formation of an additional aromatic hydrogen bond between the side chain of Tyr688 and glycine. Positioning of the carboxylate group of both ligands is similar in the wild-type protein; however, due to the absence of the guanidium group of Arg523 inside the binding pocket in the mutant receptor, there is a conformational difference between glycine and D-serine whereby the carboxylate group on glycine has a “downward” rotated orientation relative to the carboxylate group in D-serine. Meanwhile, the ammonium group is held in place by Asp732 and is comparable between both the wild-type protein and the S688Y mutant (Figure 2 and Figure 3). 

The analysis demonstrated that there was only one binding pose for glycine with a docking score of −5.28 kcal/mol, whereas there were two poses present for D-serine with a top Glide XP score of −7.37 kcal/mol. However, for the S688Y GluN1 LBD, there were four possible docked conformations for glycine, with a top docking score of −4.10 kcal/mol. There were nine output poses for D-serine with a top docking score of −3.91 kcal/mol. In both ligands, the top S688Y docking score was less negative compared to the wild-type, which can be indicative of poor ligand affinity in the receptor site.

### 2.2. Microsecond MD Analysis

To assess the stability of the systems and their behavior, we conducted 1 μs MD simulations on the apo state, as well as on top, binding poses of glycine and D-serine in wild-type and S688Y mutant receptors. Overall, the 1 μs MD simulations demonstrated that the simulation systems consisting of the solvated GluN1 LBD with glycine and D-serine ligands were stable.

**Figure 4:**
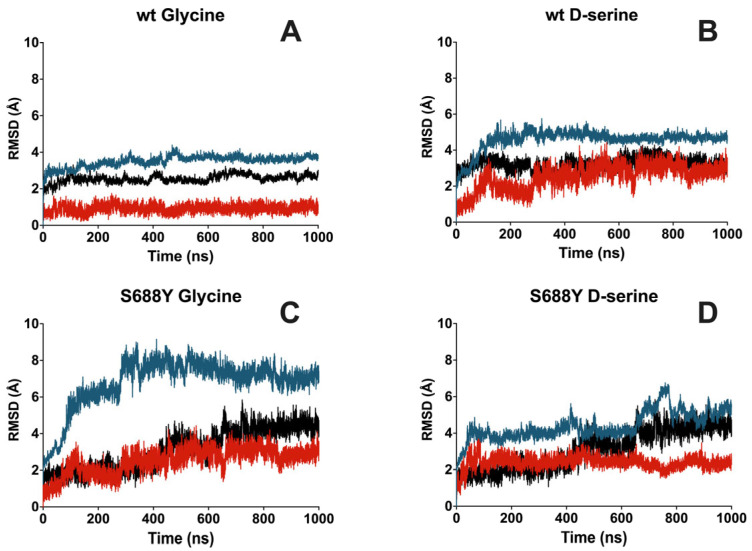
**Averaged triplicate protein and ligand RMSD for GluN1 LBD with glycine and D-serine bound over the course of 1000 ns (1 μs) molecular dynamics simulation**. RMSD values calculated for Cα are shown in blue, RMSD values calculated for residues within 5 Å of the ligand are shown in black, and ligand RMSD fit to protein is shown in red. (**A**) Wild-type with glycine. (**B**) Wild-type with D-serine. (**C**) S688Y with glycine. (**D**) S688Y with D-serine. Graphs were generated using GraphPad Prism version 9.5.0 for MacOS, GraphPad Software, San Diego, CA, USA.

The apo simulations performed demonstrated no significant fluctuations with protein RMSD fluctuating and stabilizing at approximately 4.0 Å from the input structure (Supplementary Figure S2). Overall protein RMSD in the wild-type glycine and D-serine docked poses stabilized at 3.7 Å for glycine and around 4.0 Å for D-serine as compared to the input structure (Figure 4A,B). RMSD of residues within 5 Å of the binding pocket stabilized at 2.5 Å for glycine and 3.2 Å for D-serine in the wild-type receptor pocket. The binding pocket RMSD for the S688Y receptor increases to 4.1 Å for glycine and 4.3 Å for D-serine. Furthermore, flexible loop regions within the protein are similar compared to those observed in the crystal structure [46] (Supplementary Figure S2).

Glycine ligand RMSD values, when compared against the wild-type protein, hover approximately at 1 Å while D-serine ligand RMSD in the analogous comparison is approximately 2.6 Å (Figure 4A,B). In the case of the S688Y mutation, glycine RMSD, when fitted against the protein, is approximately 3 Å, while D-serine has a ligand RMSD fitted against the protein of approximately 2.4 Å (Figure 4C,D). D-serine demonstrated two binding modes with the wild-type receptor, with contact being present with Gln686 in some frames of the trajectory (Supplementary Figure S3). Ligand–receptor interactions during the entire simulation for all four liganded simulation systems remained comparable with our observations from the docking experiments. 

**Figure 5:**
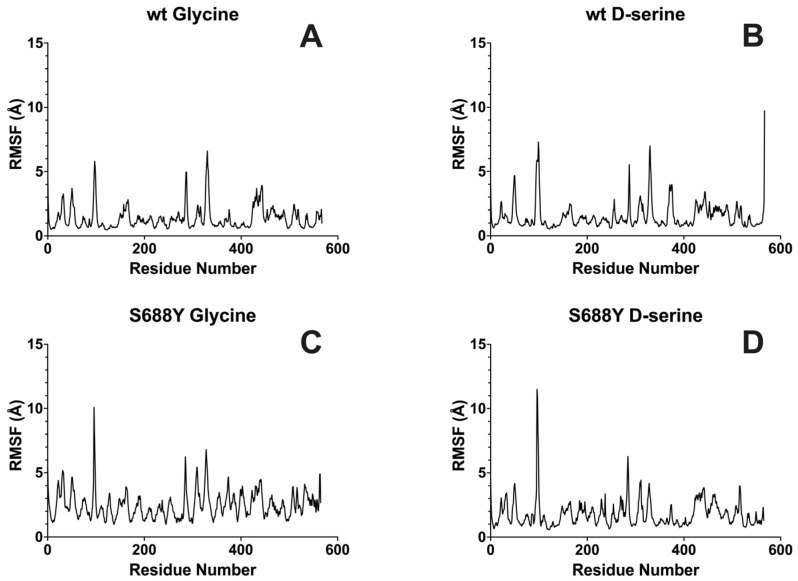
**Averaged triplicate root–mean–square fluctuation (RMSF) of the GluN1 receptor with bound ligands.** (**A**) Wild-type GluN1 with glycine bound. (**B**) Wild-type GluN1 with D-serine bound. (**C**) S688Y GluN1 mutant with glycine bound. (**D**) S688Y GluN1 mutant with D-serine bound. Graphs were generated using GraphPad Prism version 9.5.0 for MacOS, GraphPad Software, San Diego, CA, USA.

Individual residue root–mean–square fluctuation (RMSF) comparison between the wild-type and the S688Y mutation LBD shows that per-residue fluctuation increased by 27% for the glycine-bound GluN1 receptor and 10% for the D-serine-bound GluN1 receptor, respectively (Figure 5).

**Figure 6:**
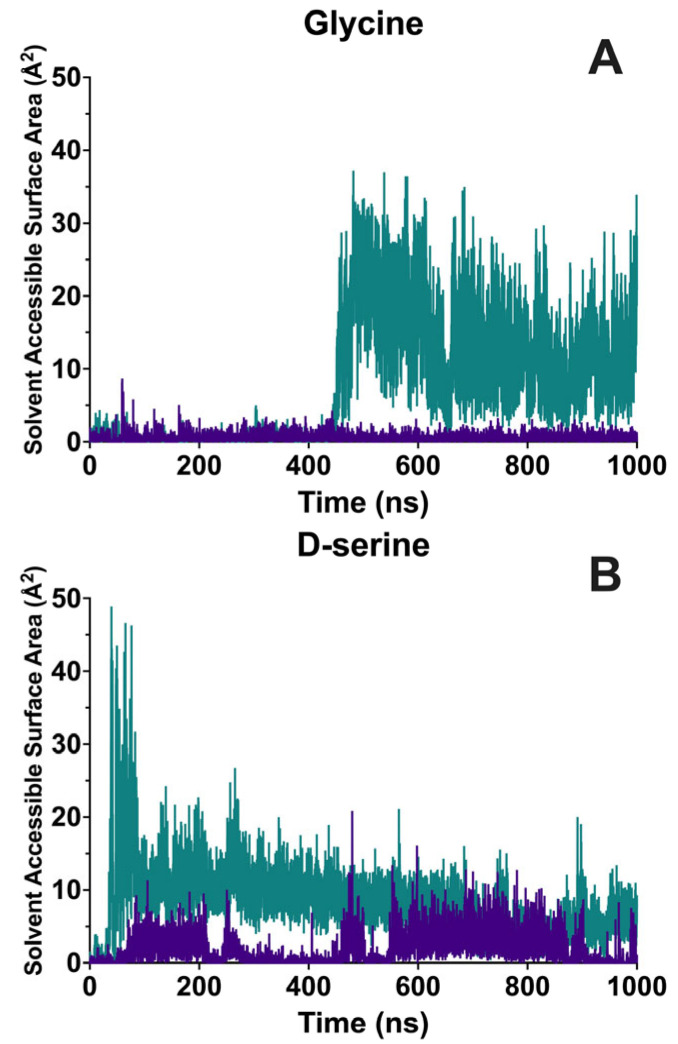
**Averaged triplicate Solvent Accessible Surface Area (SASA) of the ligand during the microsecond simulations.** Wild-type is shown in purple, and S688Y mutant receptor is shown in teal. (**A**) Glycine bound to GluN1 LBD; (**B**) D-serine bound to GluN1 LBD. Graphs were generated using GraphPad Prism version 9.5.0 for MacOS, GraphPad Software, San Diego, CA, USA.

Solvent accessibility of the ligand in the wild-type LBD during the entire course of the simulation was primarily limited to the ammonium group of glycine and D-serine. In the case of D-serine, the side chain hydroxyl group was also solvent accessible for 5% of the simulation time. Observations of a higher SASA of the ligand are indicative of the receptor providing a complex state with less solvent exposure. The solvent-accessible surface area (SASA) (Figure 6) for glycine bound to wild-type protein reached a peak of ~12 Å^2^ and stabilized in the range of 0–4 Å^2^ for most of the simulation while the SASA for D-serine bound to the wild-type protein fluctuated between ~6 Å^2^ and a more solvent accessible conformation of ~12 Å^2^.

**Figure 7:**
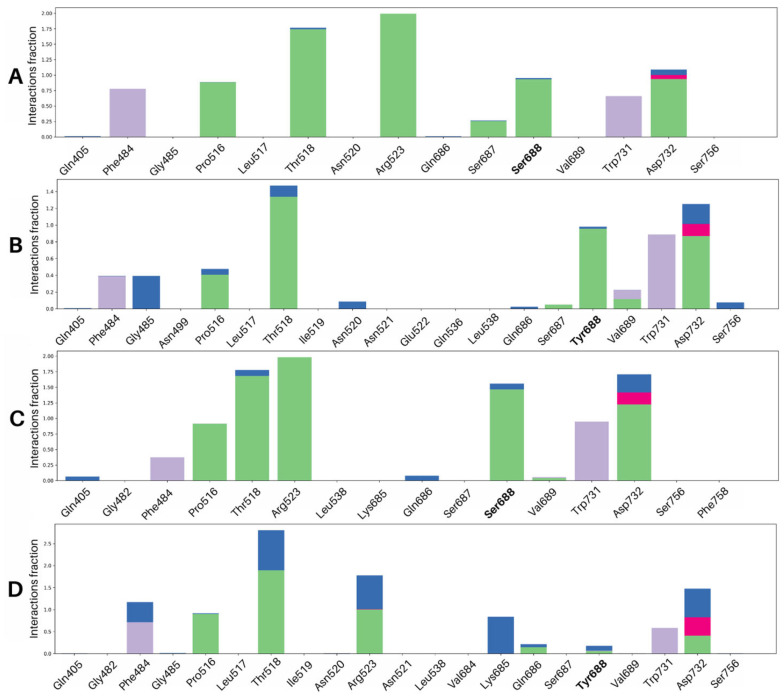
**Averaged triplicate GluN1 LBD-ligand interactions of the 1 µs simulations.** Note that figure scales are different to improve the visibility of the data. Hydrogen bonds are defined as an H-Acceptor distance less than 2.8 Å and a Donor-H-Acceptor angle greater than 120. Hydrophobic interactions include pi-pi stacking, pi-cation interactions, and van der Waals interactions within 3.6 Å of ligand. Ionic interactions are defined as charged interactions within 3.6 Å of the ligand. Water bridges are defined as H-Acceptor distances less than 2.7 Å. Hydrogen bonds are shown in green, hydrophobic interactions are shown in lilac, ionic interactions are shown in magenta, and water bridges are shown in blue. For residues with more than one type of interaction, the interaction fraction can exceed 1. (**A**) Glycine bound to wild-type GluN1 LBD, (**B**) D-serine bound to wild-type GluN1 LBD, (**C**) Glycine bound to S688Y GluN1 LBD, (**D**) D-serine bound to S688Y GluN1 LBD. Graphs were generated using the Simulation Interactions Diagram module of Maestro 2021.4 [47,48].

Analysis of the trajectories of the S688Y receptor showed that there was extensive water accessibility of the ligand compared to the wild-type protein, with solvent contact of the ligand reaching as high as 71% of the simulation time. The SASA for glycine bound to the S688Y LBD demonstrated stability around 4 Å^2^ until ~450 ns, where the loss of interactions with Thr518 and Asp732 (Figure 6 and Figure 7) led to a large increase in SASA reaching as high as 45 Å^2^. For D-serine bound to the S688Y LBD, there was an initial period of high solvent accessibility; however, SASA stabilized around 10–15 Å^2^. There were no large changes in the SASA due to changes in the Thr518 and Asp732 interactions, as they remained stable for the duration of the simulation.

Ligand contact with the guanidinium side chain of the key residue Arg523 was present in 99% of frames in the wild-type receptor. Ligand contact with Ser688 revealed a preference for the main chain nitrogen but not the side chain as seen in the docking poses with hydrogen bonds with the main chain nitrogen present for 76% of simulation time for glycine and 94% in D-serine while hydrogen bonds for the side chain were at 15% and 27% of simulation time for glycine and D-serine, respectively. Ligand interactions with the guanidium side chain of Arg523 were lost upon mutation for glycine; however, D-serine was able to maintain hydrogen bonds with the guanidium side chain of Arg523 in the S688Y GluN1 LBD.

### 2.3. Molecular Dynamics Simulations

**Figure 8:**
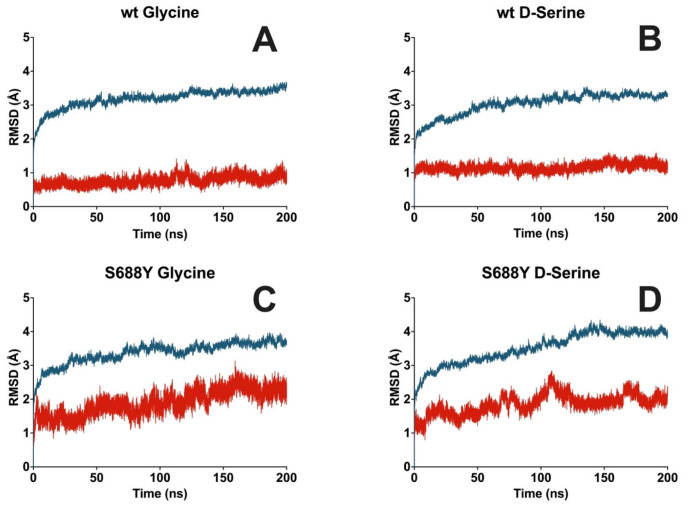
**Averaged protein and ligand RMSD for GluN1 LBD with glycine and D-serine bound over the course of quintuplet 200 ns molecular dynamics simulation**. The results shown are the average of five randomly seeded replicates. RMSD values calculated for Cα are shown in blue, and ligand RMSD fit to protein is shown in red. (**A**) Wild-type with glycine. (**B**) Wild-type with D-serine. (**C**) S688Y with glycine. (**D**) S688Y with D-serine. Graphs were generated using GraphPad Prism version 9.5.0 for MacOS, GraphPad Software, San Diego, CA, USA.

**Figure 9:**
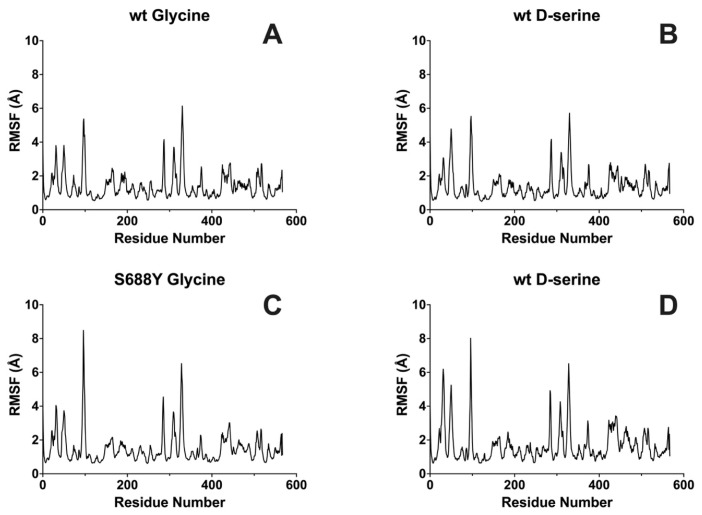
**Averaged root–mean–square fluctuation (RMSF) of the GluN1 receptor with bound ligands.** The results shown are the average of five randomly seeded replicates. (**A**) Wild-type GluN1 with glycine bound. (**B**) Wild-type GluN1 with D-serine bound. (**C**) S688Y GluN1 mutant with glycine bound. (**D**) S688Y GluN1 mutant with D-serine bound. Graphs were generated using GraphPad Prism version 9.5.0 for MacOS, GraphPad Software, San Diego, CA, USA.

To determine the reproducibility of the simulations and to potentially observe any other plausible protein–ligand interactions, we performed five 200 ns simulations that were randomly seeded to have alternative starting velocities and one that used the identical seed as in the microsecond production runs for each of the four liganded simulation systems (Supplementary Table S1). When using the same seed used in the microsecond simulations, the 200 ns simulation gave results identical to the first 200 ns of the longest production run. Compared to the microsecond simulations, the simulations with randomized initial velocities for both glycine and D-serine bound to the wild-type protein were comparable and had similar protein-ligand contacts for all simulations. Solvent contacts were present in three out of the five simulations for glycine and present in all simulations for D-serine. In the wild-type protein, all interactions observed were similar to those observed in the microsecond simulations. SASA for both glycine and D-serine stabilized around 1 Å^2^ with a maximum of 10 Å^2^ (Figure 8 and Figure 9).

In the S688Y mutated protein, solvent exposure was observed in all simulations with both glycine and D-serine. Both glycine and D-serine bound to the S688Y mutated protein experienced an average SASA in the range of 15–30 Å^2^ for all seeds, contrasting with observations of the wild-type protein, which showed SASA in the range of 4 Å for glycine and 7 Å for D-serine. Interestingly, seed 1293 of glycine with S688Y mutated protein demonstrated a complete dissociation of glycine from the receptor binding site. 

### 2.4. Binding Free Energy Calculations

To estimate the binding free energy of glycine and D-serine, we performed MM-GBSA free energy calculations to approximate the ΔG_bind_ to both the wild-type and S688Y mutant receptors for an estimation of the relative overall binding affinity of the ligand to the receptor and the individual components (Table 1). 

Compared to the wild-type receptor, the calculated binding free energy in the S688Y mutant receptor was increased from −30.0 kcal/mol to −26.3 kcal/mol for glycine and −34.6 kcal/mol to −22.7 kcal/mol for D-serine. This result is directly comparable to previous electrophysiology studies on the S688Y mutant, where the EC_50_ of the ligands was increased significantly compared to the native receptor [33]. The contribution from electrostatic interactions (ΔG_bind_ coulomb) increased from −5.62 kcal/mol to 11.8 kcal/mol for glycine and −6.62 kcal/mol to 7.58 kcal/mol for D-serine. This strongly supports that it is no longer energetically favorable for the system to form salt bridges which are consistent with our results from the MD trajectories where electrostatic interactions between the ligand and Arg523 were present in far fewer frames in the S688Y protein compared to the wild-type, especially for the glycine ligand where Arg523 interactions were almost completely absent. There were minor changes to other binding affinities, which included an increased contribution from hydrogen bonding (ΔG_bind_ hydrogen bonding) in both glycine and D-serine post-mutation, which is reflective of our docking results. Lipophilic interactions (ΔG_bind_ lipophilicity) decreased slightly for glycine, which reflects our previous observations where the ligand environment is slightly more hydrophobic (Figure 2A,C); however, this did not change significantly for D-serine.

Solvent interactions (ΔG_bind_ solvent) became more favorable, changing from −7.04 kcal/mol to −18.9 kcal/mol for glycine and similarly a drop from −6.18 kcal/mol to −9.65 kcal/mol for D-serine, which reflects our observations from the simulations where there was a significant increase in solvent exposure in the mutated protein. This greater extent of ΔG_bind_ solvent decrease for glycine is also consistent with our observations where complexes containing glycine had a higher number of water bridges in the S688Y receptor compared to D-serine, which had a modest increase. Van der Waals interactions (ΔG_bind_ van der Waals) also decreased for glycine from −9.97 kcal/mol to −13.2 kcal/mol and −13.8 kcal/mol to −14.9 kcal/mol for D-serine. This may be due to the change in the environment around the glycine to one which is more hydrophobic.

## 3. Discussion

In the microsecond simulations of the NMDAR ligand binding domain and ligand, there was a demonstrable increase in the presence of water bridges interacting with both glycine and D-serine in the mutated receptor containing the S688Y mutation compared to the wild-type. A group of five water molecules, normally in contact with the ligand [7], is involved in binding for ligands at the GluN1 LBD. This involvement is observed in our microsecond simulations of wild-type protein with both glycine and D-serine (Figure 7A,B). However, these water molecules do not appear to contribute significantly to the wild-type binding network compared to the S688Y mutant. D-serine further demonstrates two possible poses it could use to bind to the wild-type receptor (Figure 3), including an extra contact with Gln686 in the second pose. In the mutated receptor, the water in contact with the ligand increased (Figure 7C,D) relative to that in the wild-type receptor. There was also instability observed with the SASA of the ligands, where values hovered around 5–10 Å for both glycine and D-serine in the wild-type receptor but increased significantly in the mutated receptor. This is important to note as the bulk water in contact water in contact with the ligand can behave differently from structured, isolated water within the binding site [49] and instead contribute to the instability of the system. Furthermore, large protein RMSD differences were observed especially in the case of mutated receptors, reaching 8 Å (Figure 4C,D) compared to those in the wild-type receptor, which fluctuated around 4.5 Å (Figure 4A,B). The present study focuses on the simulation of the LBD in isolation and the interactions of the ligand with the LBD. This approach is appropriate for the present implementation, as shown by Wang et al., (2021) [47], where the LBD and transmembrane domain (TMD) are linked, while each of the subunits remains independent and is able to maintain their structure throughout the transition process.

Loss and weakening of key interactions are also observed in the simulations, which contribute to an increase of ΔG_bind_ and especially the key interaction of the guanidium group Arg523 with the ligand. This interaction is absent in the S688Y receptor with glycine in the microsecond simulation and present in fewer than 20% of frames in the random-seeded simulations. The loss of this key interaction, combined with the change in the binding pocket, contributed to the increase in ΔG_bind_ for both glycine and D-serine. In the current study, we performed the MM-GBSA calculations on the final 50 ns of the stable 200 ns trajectories; however, it would be of interest for future studies to determine whether a calculation over a longer trajectory yields similar results to the findings presented here.

Results from the ΔG_bind_ calculations show that there is a significant decrease in binding affinity for the S688Y mutant LBD compared to that of the wild-type protein with an increase of 11.9 kcal/mol in ΔG_bind_ for D-serine and an increase of 3.75 kcal/mol in ΔG_bind_ for glycine. This is of particular importance due to GluN1/GluN2A’s higher affinity for D-serine compared to glycine in the forebrain regions [48] and that synaptic NMDARs are gated by D-serine [46,50,51,52]. ΔG_bind_ coulomb values increase to 11.83 kcal/mol and 7.58 kcal/mol for glycine and D-serine, respectively, upon mutation, showing that the interference of Tyr688 between the guanidium group of Arg523 and the ligand was able to disrupt this key interaction present in the wild-type NMDA GluN1 LBD. However, the rotamer state for Tyr688 in this study differs from those previously reported [33]. This difference leads to a change in conformation for other residues in the receptor binding site and is significant due to the interference with Arg523. Though we have run substantive molecular dynamics calculations, which should sample favorable conformations, a further study to confirm the extent of any biases from the initial side chain conformation of the receptor model should also be performed. Especially for Tyr688, due to the multiple different conformations, the residue side chain could adapt and impact the surrounding receptor environment. Due to the small size of the glycine and D-serine ligands, this observed difference is potentially very significant for understanding the binding of the ligand. MM-GBSA calculations are suitable for the present scoping study as an initial starting point for the understanding of ligand binding affinity of the endogenous glycine and D-serine ligands to the NMDA GluN1 LBD. Implicit solvent-based approximation methods have been shown to perform well [53] compared to explicit solvent models such as FEP. Future work will employ the implementation of free energy perturbation (FEP) [54,55], which is significantly more complex but will result in ligand binding calculations with much greater confidence.

Traditionally, MD simulations are carried out on the nanosecond scale; however, we have performed microsecond simulations which gives us confidence that the possible ligand poses are well sampled, and this, combined with randomly seeded 200 ns simulations, helps to overcome issues of interpretation from reproducibility of MD simulations and has ensured that the initial system is stable under multiple conditions. Therefore, we are confident in our approach of using the refined docked structure as a starting point for the simulation. The use of the OPLS3e force field is widespread in docking [56,57]; however, due to limitations regarding local hardware and software implementations, the OPLS_2005 force field was used for the current protocol. Future protocols will take advantage of the latest OPLS4 force field. Our choice of the SPC water model is appropriate, despite the potential advantages of other models [58,59]. Furthermore, future studies should integrate simulations of the full NMDAR combined with in vitro analysis. 

Our docked poses agree with previously published X-ray structures and show that this approach is suitable for studying the detailed changes which may occur between the wild-type and mutated receptor. The use of the XP scoring function allows the elimination of false positive docking poses to ensure confidence in the generated poses. Furthermore, differences in docking scores between the wild-type receptor and the S688Y receptor reported by the XP scoring function correlate with the calculated free energy of binding from the MD simulation trajectories. The current study looks at one of the currently known mutations within the NMDAR GluN1 LBD; however, other possible mutations within the LBD and in regions in proximity could influence the binding of ligands to this receptor in other ways. Therefore, future studies should aim to ascertain the effects these other mutations have on the LBD and ligand affinity to the binding site. Docking was selected over insertion for ligand placement as binding may not be identical in the S688Y mutant LBD compared to the wild-type, and the use of docking for both parts ensures that there is consistency and comparison between the two systems. As shown by our docking results, the output from glycine and D-serine docking to the wild-type receptor is comparable to those observed in the X-ray crystallography structures as determined by Furukawa and Gouaux [7].

From our results, the S688Y mutation leads to poor binding of both glycine and D-serine to the NMDAR GluN1 subunit. This may result in partial or non-activation of the receptor, which results in issues during early development, which may contribute to an explanation on the molecular level of the encephalopathies as described by Zehavi et al. [34] and further characterized by Skrenkova et al. [33] in vitro where the mutation of the receptor resulted in a drop in EC_50_ of both D-serine and glycine and also reduced cell surface trafficking of the NMDAR. This manuscript outlines the two primary endogenous ligands for the NMDAR GluN1 subunit, D-serine, and glycine, as characterized by Skrenkova et al. [33]. However, other endogenous ligands, such as L-aspartate, and antagonists, such as 5,7-dichlorokynurenic acid, could also be studied in future studies. 

## 4. Materials and Methods

Figures in this paper were prepared using PyMOL [44] and the Maestro visualization interface [45]. Calculations were performed on Intel Xeon E5-2680 V3 CPUs and Nvidia V100 SXM2 GPUs. Graphs were generated using the Simulation Interactions Diagram module in Desmond 2018-4 [45,60] and GraphPad Prism version 9.5.0 for MacOS, GraphPad Software, San Diego, CA, USA.

### 4.1. Receptor and Ligand Preparation

Criteria for model selection include the absence of allosteric modulators, the absence of stabilizing mutations or linkers, high-resolution structures, and the absence of large inhibitors, which may influence the structure of the LBD directly. Therefore, the crystal structure of the GluN1/GluN2A ligand-binding domain in complex with glycine and glutamate (PDB: 4NF8) [61] was selected due to its high resolution of 1.86 Å and absence of allosteric modulators or large inhibitors.

The structure was downloaded and prepared using the protein preparation module in Schrödinger Maestro [45,62]. In brief, this involved assignment of bond orders, adding missing hydrogens, and connection of disulfide bonds. Protonation states were assigned corresponding to pH 7.0 ± 2.0 using PROPKA [63,64]. All water molecules in the PDB entry were removed, and the protein structure was minimized with positional restraints as per default settings using the OPLS3e force field [65]. 

The S688Y mutation was introduced at the GluN1 binding site using the Maestro residue mutation interface and the rotamer with the highest standard population and probability based on their respective dihedral angles determined using the rotamer selection tool. This corresponds to a chi1 of −66° and chi2 of 98°. Minimization was subsequently carried out with position restraints on Cα atoms using the VSGB solvation model with the OPLS3e force field [65].

### 4.2. Molecular Docking

Glycine and D-serine were docked into the wild-type and mutant GluN1 binding pockets in their fully ionized form using the Glide docking module [45,66,67,68] with flexible ligand sampling and the extra precision (XP) scoring function. Van der Waals radii were scaled by a factor of 0.80, and a partial charge cut-off of 0.15 eV for non-polar ligand atoms was applied. The receptor grid to guide the docking (Supplementary Figure S1) was defined using the co-crystallized glycine from the original PDB structure by Furukawa and Gouaux [7] as the centroid of the receptor site and therefore, the receptor grid for docking with a boundary of 20 Å × 20 Å × 20 Å. The van der Waals, radius scale factor, was set to 1.0, and a partial charge cut-off to 0.25 eV. The maximum number of conformations and output per ligand was set to 10. Glide utilizes a relative docking energy for each docking pose, with lower energies being more favorable. Docking was carried out using a flexible receptor sampling algorithm to ensure receptor residue conformations were sampled.

### 4.3. Molecular Dynamics (MD) Simulation

The top binding pose for each of the ligand/receptor combinations was subjected to molecular dynamics (MD) simulations for further analysis. Simulation systems were built using the System Builder tool in Desmond 2018-4 [45,60] using the OPLS_2005 force field, suitable for local hardware [69], and a cubic system with a 10 Å × 10 Å × 10 Å buffer around the protein. Each system was solvated using the explicit SPC water model [59,70]. The overall charge of the system was neutralized by substituting water molecules with Cl^−^ and further NaCl was added to a concentration of 0.15 M by random replacement of water molecules. Each system was subject to the default relaxation protocol, which involved the relaxation of the simulation system using default NVT and NPT ensembles; for more detail regarding the minimization and equilibration code, please see Supplementary Code S1. 

Simulations were conducted for each system using identical starting parameters, differing only in the seed values of the randomized initial starting velocities. Simulations were performed for a period of 1 μs with a sampling interval of 20 ps and a default seed of “2007”. Subsequent simulations were 200 ns in duration with a sampling interval of 5 ps using the default seed of “2007” and five randomized seeds for the calculation of initial velocities for the MD simulations (Supplementary Table S1). Simulation trajectories were subsequently analyzed using the simulation interaction analysis interface of Desmond [47,50] to determine the RMSD, RMSF, and SASA values for the receptor and ligand of interest.

### 4.4. Binding Free Energy Calculations

The final 50 ns from the 200 ns MD trajectories (fixed seed “2007”) were used to estimate the affinity of glycine and D-serine for the GluN1 and mutant receptor using the generalized Born and surface area (MM-GBSA) [71,72] method. The Gibbs binding free energy (Δ*G_bind_*) was computed based on the equation shown below:

$$\Delta G_{bind}=G_{complex}-G_{protein}-G_{ligand}$$

Δ*G_bind_* is a measure of estimating the binding affinity of a ligand to a protein, with negative values corresponding to energetically favorable reactions and a positive value being energetically unfavorable.

The MM-GBSA calculations were performed using the thermal_mmgbsa.py plugin of Prime [73,74] as supplied [45]. Calculations were performed using the OPLS3e [65] force field and the polarisable variable-dielectric Generalised Born (VSGB) 2.1 implicit solvation model, which has been thoroughly validated [75]. In brief, the script extracts the final 50 ns and performs geometry optimization to extract energies of the complex, the protein, and the ligand to calculate Δ*G_bind_*. MM-GBSA is a method for predicting the binding energy of protein–ligand complexes. This method is based on several approximations, including the molecular mechanics (MM) approximation for describing the atomic interactions, the Generalised Born (GB) approximation for modeling solvent effects, and the surface area (SA) approximation for calculating the non-polar contribution to the binding energy. Despite these approximations, MM-GBSA is a valuable tool for rational drug design that provides a good balance between computational cost and accuracy.

## 5. Conclusions

Early infantile epileptic encephalopathies are a debilitating group of conditions that have multiple causes. The existence of mutations within the NMDAR GluN1 subunit is well documented in the literature; however, mutations within the LBD have only been discovered recently, and a complete understanding of the impacts of these mutations on receptor function is yet to be achieved. Our current research demonstrates how computational drug discovery tools, including ligand docking and extended molecular dynamics simulations, can be used to bridge this gap. Our results show that the S688Y mutation of the NMDA GluN1 receptor is detrimental to ligand binding and affinity, particularly for the binding of D-serine. This may provide an explanation for some of the clinical observations [2]. In future studies, simulation of the full-length receptor similar to those performed by Černý et al. [30] and Sinitskiy and Pande [32], as well as experimental determination of the mutated S688Y receptor structure would also be welcome, as would other mutant NMDAR structures, to assist the study of the available ligand binding modes, and ultimately the design of agents that can modulate the activity of mutant receptors to aid personalized treatments.

The present study presents an insight into the impact of the S688Y mutation on NMDAR GluN1 subunit function and associated endogenous ligand affinity, particularly glycine and D-serine. Clinically, this study contributes to one aspect regarding the underlying molecular mechanisms, which may lead to symptoms associated with severe early infantile encephalopathy and associated symptoms such as intellectual disability [34,38], epilepsy [35], and postnatal microcephaly [76]. Further understanding of the implications of mutations within the LBD of the NMDA GluN1 subunit may aid in the development of personalized treatments based on patient genotyping and may be picked up during genetic screening during pregnancy [77].

## Figures and Tables

**Table 1:** Binding free energy, ΔG_bind,_ values for glycine and D-serine bound to the wild-type and S688Y mutant receptor. The values are averaged values of the five randomly seeded replicates.

System		ΔG_bind_	ΔG_bind_ Coulomb	ΔG_bind_ Covalent	ΔG_bind_ H-Bond	ΔG_bind_ Lipophilicity	ΔG_bind_ Solvent	ΔG_bind_ Van der Waals
wt Glycine	Mean (kcal/mol)	−30.0	−5.62	0.09	−5.05	−2.43	−7.04	−9.97
	Standard Deviation	3.08	4.27	0.42	0.54	0.15	3.56	2.64
wt D-serine	Mean	−34.6	−6.62	1.09	−5.00	−4.03	−6.18	−13.8
	Standard Deviation	3.21	4.90	0.54	0.36	0.18	3.47	2.45
S688Y Glycine	Mean	−26.3	11.8	−0.11	−3.08	−2.80	−18.9	−13.2
	Standard Deviation	1.94	3.44	0.59	0.26	0.10	3.33	1.78
S688Y D-serine	Mean	−22.7	7.58	0.84	−3.32	−3.18	−9.65	−14.9
	Standard Deviation	2.03	3.42	0.63	0.29	0.31	2.92	1.32

## Data Availability

Simulation data and other materials will be provided upon request.

## Supplementary Materials

The following supporting information can be downloaded at: https://www.mdpi.com/article/10.3390/molecules28104108/s1, Table S1: Seed values of randomly seeded MD simulations. Figure S1: Position of the ligand docking grid; Figure S2: Trajectory analysis for apo state NMDA ligand binding domain during 1 μs molecular dynamics simulation; Figure S3: Ligand–protein interaction timeline during the 1000 ns (1 μs) simulation; Code S1: Relaxation protocol for Desmond.

## Sample Availability

Samples of the compounds are not available from the authors.
