# Supplementary material for: Binding and Dynamics Demonstrate the Destabilization of Ligand Binding for the S688Y Mutation in the NMDA Receptor GluN1 Subunit
Source: Molecules. 2023 May 15;28(10):4108. doi: 10.3390/molecules28104108 (PMC10224558; doi:10.3390/molecules28104108)
Supplement: Supplementary file 1 [file molecules-28-04108-s001.zip › molecules-2357070-supplementary.pdf]

Supplementary information

Binding and Dynamics Demonstrate the Destabilization of Ligand Binding for the S688Y Mutation in the NMDA Receptor GluN1 Subunit

Jake Zheng Chen [1,2], William Bret Church [1], Karine Bastard [1], Anthony P. Duff [3] and Thomas Balle [1,2,*]

1 Sydney Pharmacy School, Faculty of Medicine and Health, The University of Sydney, Camperdown, NSW 2006, Australia; bret.church@sydney.edu.au (W.B.C.)
2 Brain and Mind Centre, The University of Sydney, Camperdown, NSW 2050, Australia
3 National Deuteration Facility, Australian Nuclear Science and Technology Organization, New Illawarra Road, Lucas Heights, NSW 2234, Australia
* Correspondence: thomas.balle@sydney.edu.au

Corresponding residue numbering in supplementary information is as follows:

Q13=Q405, G90=G482, F92=F484, G93=G485, N107=N499, P124=P516, L125=L517, T126=T518, I127=I519, N128=N520, N129=N521, E130=E522, R131=R523, Q144=Q536, L146=L538, V176=V684, K177=K685, Q178=Q686, S179=S687, S180=S688/Y180=Y688, V181=V689, W223=W731, D224=F732, S248=S756, F250=F758

Table S1. Seed values of randomly seeded MD simulations. A total of 6 200 ns simulations were carried out for each ligand-protein complex which includes one simulation using a standardised seed 2007 and 5 randomly seeded simulations.

	**Wild-type Glycine**	**Wild-type D-serine**	**S688Y Glycine**	**S688Y D-serine**
Random 1	9635	4818	1293	0548
Random 2	1868	5136	6963	9443
Random 3	9881	0776	7824	3612
Random 4	3369	9099	4219	5870
Random 5	1671	5332	8257	9304

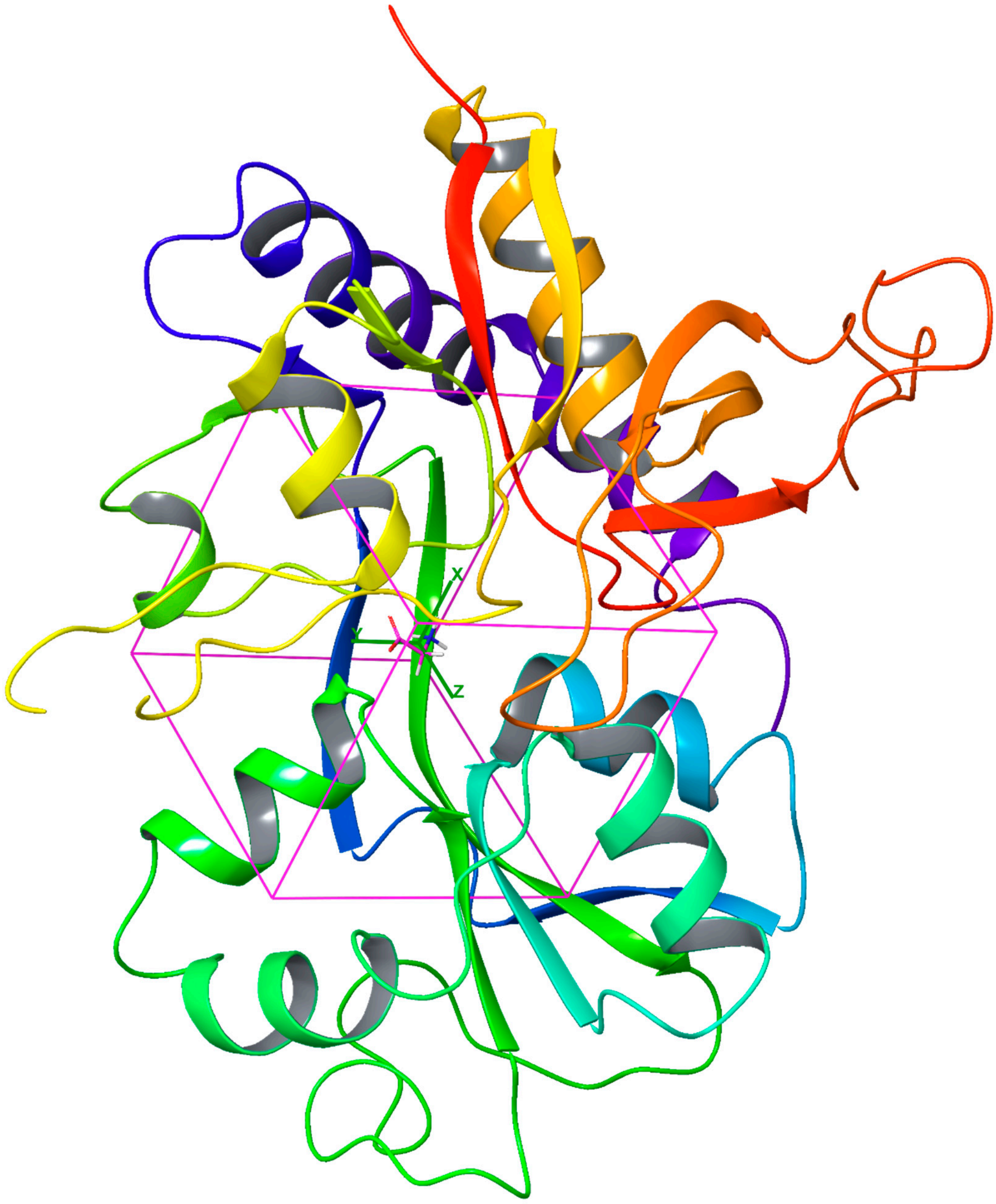

Figure S1. Position of the ligand docking grid. 20 Å x 20 Å x 20 Å (purple box) superimposed on the NMDAR GluN1 LBD (PDB: 4NF8) shown in cartoon style. Glycine is shown in magenta at the centre of the box and the XYZ axes are shown in green at the centre of the docking grid. Colouring of the cartoon is according to the protein sequence from red to purple.

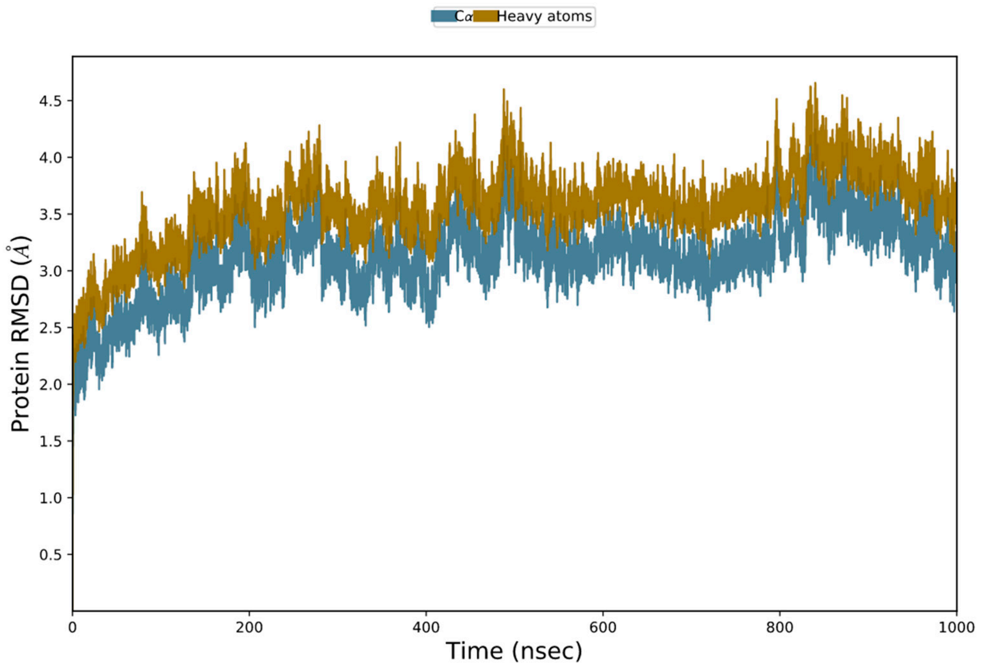

A

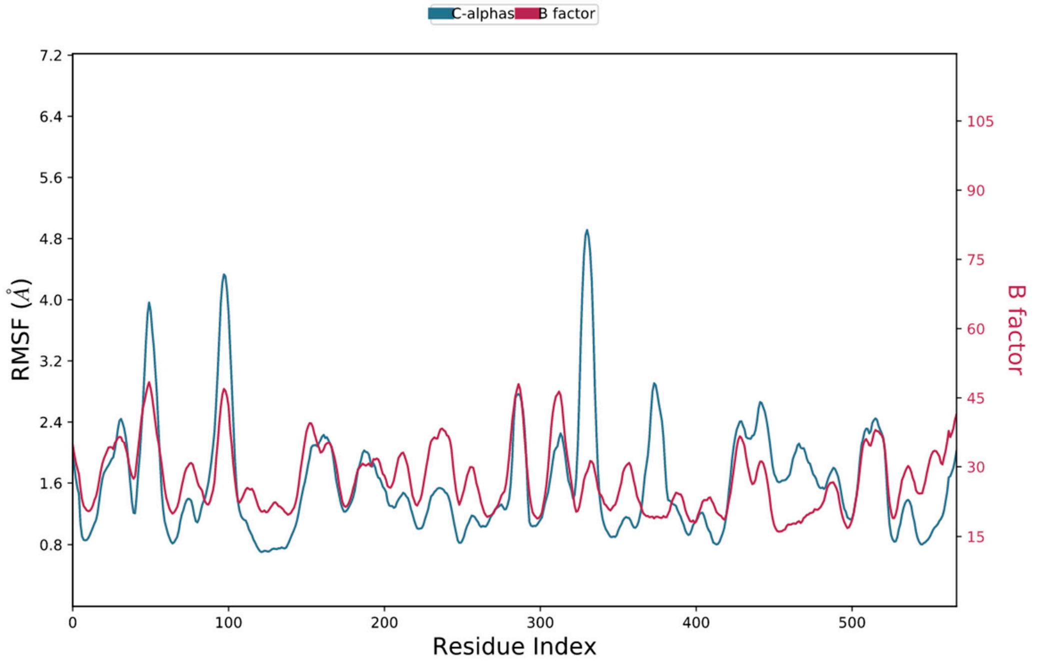

B

Figure S2. Trajectory analysis for apo state NMDA ligand binding domain during the 1 μs molecular dynamics simulation. (**A**) RMSD plot of the protein over the length of the trajectory, the protein stabilised around 3.5 Å from the starting structure. (**B**) Root mean square fluctuation (RMSF) plot per residue. Regions with

higher flexibility matches those in the crystal structure. Graphs generated using the Simulation Interactions Diagram module of Maestro 2021.4 [47,48].

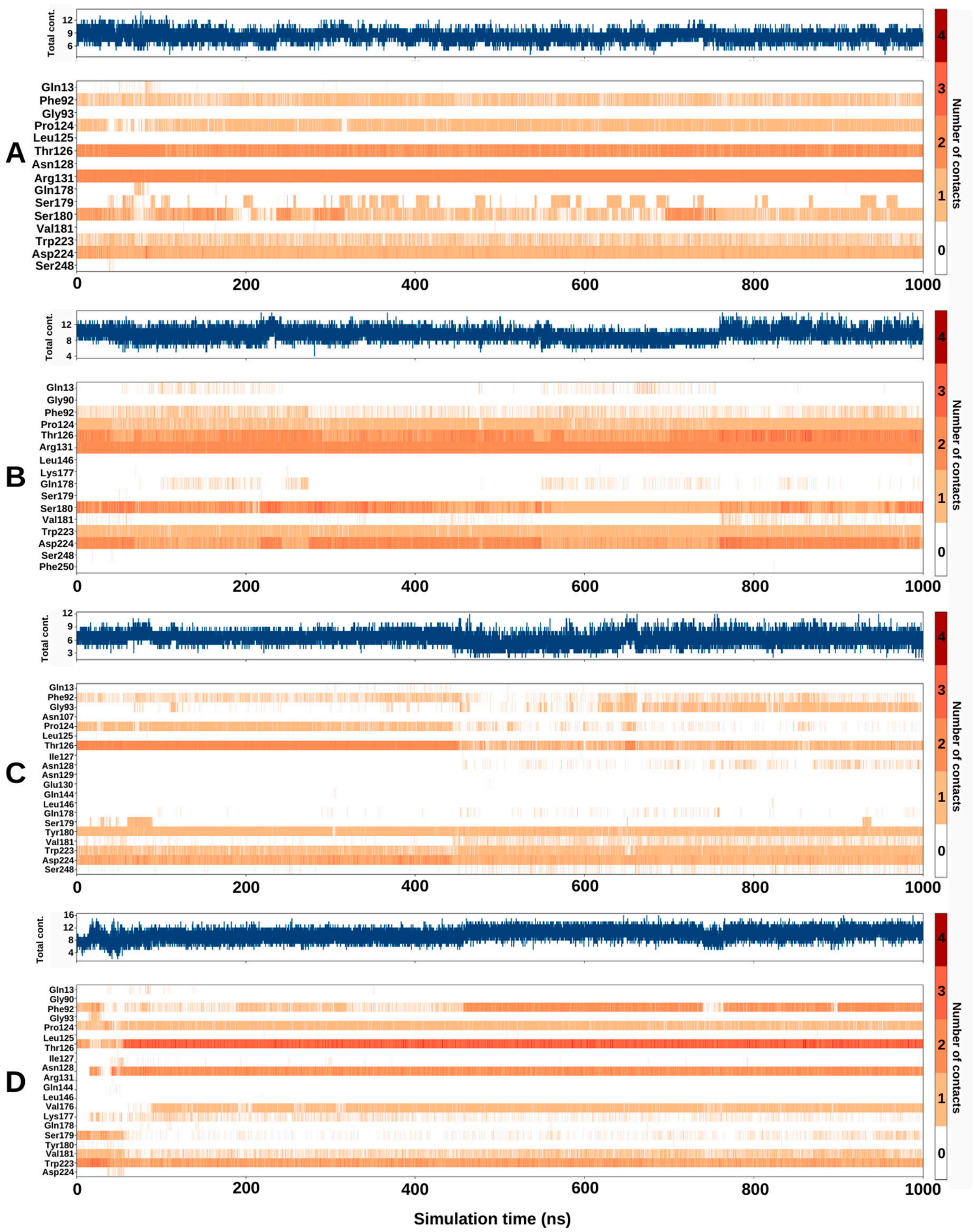

Figure S3. Ligand-protein interaction timeline during the 1000ns (1μs) simulation. Interactions consisting of a combination of hydrogen bonds, hydrophobic interactions, ionic interactions, and water bridges. Hydrogen bonds are defined as an H-Acceptor distance less than 2,8 Å, and a Donor-H-Acceptor angle of greater than 120. Hydrophobic interactions include pi-pi stacking, pi-cation interactions and van der Waals interactions within 3.6 Å of ligand. Ionic interactions are defined as charged interactions within 3.6 Å of the ligand. Water bridges are defined as H-Acceptor distance less than 2,7 Å. Top panel for each timeline tracks the sum of interactions the ligand makes with the protein over the course of the simulation. Darker red colours represent the presence of more than 1 interaction with the specific residue. (**A**) Wild type GluN1 LBD with glycine. (**B**) Wild type GluN1 LBD with D-serine. (**C**) S688Y GluN1 LBD with glycine. (**D**) S688Y GluN1 LBD with D-serine. Graphs generated using the Simulation Interactions Diagram module of Maestro 2021.4 [47,48].

Supplementary Code S1 Relaxation protocol for Desmond Relaxation protocol as used for all simulations.

```
task {
   task = "desmond:auto"
   set_family = {
      desmond = {
         checkpt.write_last_step = no
      }
   }
}

simulate {
```

```
   title       = "Brownian Dynamics NVT, T = 10 K, small timesteps, and
restraints on solute heavy atoms, 100ps"
   annealing   = off
   time        = 100
   timestep    = [0.001 0.001 0.003 ]
   temperature = 10.0
   ensemble = {
      class = "NVT"
      method = "Brownie"
      brownie = {
         delta_max = 0.1
      }
   }
   restrain = {
      atom = "solute_heavy_atom"
      force_constant = 50.0
   }
}


simulate {
   effect_if   = [["==" "-gpu" "@*.*.jlaunch_opt[-1]"] 'ensemble.method =
Langevin']
   title       = "NVT, T = 10 K, small timesteps, and restraints on solute
heavy atoms, 12ps"
   annealing   = off
   time        = 12
   timestep    = [0.001 0.001 0.003]
   temperature = 10.0
   restrain    = { atom = solute_heavy_atom force_constant = 50.0 }
   ensemble    = {
      class  = NVT
      method = Berendsen
      thermostat.tau = 0.1
   }

   randomize_velocity.interval = 1.0
   eneseq.interval             = 0.3
```

```
   trajectory.center          = [ ]
}
simulate {
   title       = "NPT, T = 10 K, and restraints on solute heavy atoms,
12ps"
   effect_if   = [["==" "-gpu" "@*.*.jlaunch_opt[-1]"] 'ensemble.method =
Langevin']
   annealing   = off
   time        = 12
   temperature = 10.0
   restrain    = retain
   ensemble    = {
      class  = NPT
      method = Berendsen
      thermostat.tau = 0.1
      barostat  .tau = 50.0
   }

   randomize_velocity.interval = 1.0
   eneseq.interval             = 0.3
   trajectory.center           = []
}

solvate_pocket {
   should_skip = true
   ligand_file = ?
}

simulate {
   title       = "NPT and restraints on solute heavy atoms, 12ps"
   effect_if   = [["@*.*.annealing"] 'annealing = off temperature =
"@*.*.temperature[0][0]"'
                  ["==" "-gpu" "@*.*.jlaunch_opt[-1]"] 'ensemble.method =
Langevin']
   time        = 12
   restrain    = retain
   ensemble    = {
      class  = NPT
```

```
      method = Berendsen
      thermostat.tau = 0.1
      barostat  .tau = 50.0
   }

   randomize_velocity.interval = 1.0
   eneseq.interval             = 0.3
   trajectory.center           = []
}

simulate {
   title       = "NPT and no restraints, 24ps"
   effect_if   = [["@*.*.annealing"] 'annealing = off temperature =
"@*.*.temperature[0][0]"'
                  ["==" "-gpu" "@*.*.jlaunch_opt[-1]"] 'ensemble.method =
Langevin']
   time        = 24
   ensemble    = {
      class  = NPT
      method = Berendsen
      thermostat.tau = 0.1
      barostat  .tau = 2.0
   }

   eneseq.interval   = 0.3
   trajectory.center = solute
}

simulate {
   cfg_file = "4nf8_apo_1us.cfg"
   jobname  = "$MASTERJOBNAME"
   dir      = "."
   compress = ""
}
```
